# Special Section Guest Editorial: Hybrid Photonic/X Neurointerfaces

**DOI:** 10.1117/1.NPh.9.3.032201

**Published:** 2022-09-30

**Authors:** Hui Fang, Xin Yu

**Affiliations:** a Dartmouth College, Hanover, New Hampshire, United States; b MGH/HST Martinos Center for Biomedical Imaging, Charlestown, Massachusetts, United States

## Abstract

The article introduces the Special Section on Hybrid Photonic/X Neurointerfaces for Neurophotonics Volume 9 Issue 3.

Brain studies have evolved in both anatomical and functional aspects. Photonic imaging has served as the main drive to study brains over a century ago since the identification of diversified neuronal morphology pioneered by Cajal and others. Given the evolved understanding of the brain, the demanding scientific needs have led to the emergence of multiple novel neurotechniques. The combination of photonics and emerging neurotechniques has opened new avenues for recording and manipulation of neuronal activity. Multimodal approaches have extended the spatiotemporal scale, resolution, and specificity achievable by each modality alone, enabling the application of these hybrid tools to studies of the brain in health and disease. This special section of *Neurophotonics* Volume 9 Issue 3 introduces twelve outstanding articles covering a broad range of hybrid photonic/X neurointerface tools to image, record, and manipulate brain structure and function across animal species and in humans.

Many of the papers feature a trend of effort in combining the global functional mapping method, e.g., functional MRI, with optical imaging or fiber photometry based on the advanced genetically encoded biosensors with optogenetic manipulation. Chao et al.^1^ have computed hemodynamic response function based on the concurrent spectral fiber-photometry and fMRI data, enabling a better understanding of the basis of fMRI signals. Lambers et al. (issue cover)^2^ have used the MR-derived hemodynamic parameters to correct the fiber-based lactate recordings with fluorescence resonance energy transfer sensors. Ioanas et al.^3^ provide a focused review on the capabilities and limitations of optical/fMRI method to study neurovascular coupling (NVC) and neuronal network mapping. Guilbert et al.^4^ provide a thorough review to cover the vascular and neural network interaction underlying the functional connectivity detected by resting-state fMRI. Cleppien et al.^5^ provide a comprehensive review to introduce the OPTO-MAgnetic Integration Concept (OPTOMAIC) to extract the temporal characteristics of the BOLD response based on optically recorded neuronal signals. Yin et al.^6^ have created a step-by-step protocol to co-implant an ultraflexible microelectrode array with a glass cranial window within a single surgery to enable simultaneous electrophysiological and optical measurements.

An additional frontier area is in the integration of photonics with ultrasound brain stimulation. Lee et al.^7^ have developed a transparent acoustic transducer based on a glass coverslip to enable simultaneous ultrasound neuromodulation and microscopic monitoring of neural responses in vivo. Shi et al.^8^ review recent developments in a variety of optoacoustic platforms for neural modulation.

Another set of papers highlights the system level considerations for multimodal approaches. Beloate and Zhang^9^ present a review to discuss the region-, cell-type-, and projection-specific manipulation and neuronal activity measurement and their linkage with global brain signaling and behavior. Meyer-Baese et al.^10^ provide a detailed review of different functional spatiotemporal dynamic patterns that have been identified by electrophysiological recordings, optical imaging, functional magnetic resonance imaging, and electroencephalography. The review by Xu et al.^11^ focuses on the mapping of substance diffusion through the brain extracellular space (ECS) in brains with multi-modal neuroimaging techniques across different scales. Lake and Higley^12^ review progress in multiphoton imaging, single-photon imaging, and fiber photometry as well as magnetic resonance imaging, and discuss their pairwise combinations with regard to the definition and study of brain networks.

Now is an exciting time for the field of neurophotonics. The capabilities of complementary neural interfacing methods have caught up and are stronger than ever. Hybrid and integrated methods are enabling and needed approaches to study the brain across all spatial and temporal scales. These studies will likely play a critical role in the development of new theoretical frameworks and the discovery of novel fundamental principles. As a community, we are poised to develop and apply convergent approaches and work aggressively toward our ultimate goal – understanding the brain.
